# Alterations in Peripheral Organs following Combined Hypoxemia and Hemorrhagic Shock in a Rat Model of Penetrating Ballistic-Like Brain Injury

**DOI:** 10.1089/neu.2019.6570

**Published:** 2020-02-04

**Authors:** Bernard S. Wilfred, Sindhu K. Madathil, Katherine Cardiff, Sarah Urankar, Xiaofang Yang, Hye Mee Hwang, Janice S. Gilsdorf, Deborah A. Shear, Lai Yee Leung

**Affiliations:** ^1^ Brain Trauma Neuroprotection and Neurorestoration Branch, Center for Military Psychiatry and Neuroscience, Walter Reed Army Institute of Research (WRAIR), Silver Spring, Maryland.; ^2^ Department of Surgery, Uniformed Services University of the Health Sciences, Bethesda, Maryland.

**Keywords:** hemorrhagic shock, hypoxemia, polytrauma, traumatic brain injury

## Abstract

Polytrauma, with combined traumatic brain injury (TBI) and systemic damage are common among military and civilians. However, the pathophysiology of peripheral organs following polytrauma is poorly understood. Using a rat model of TBI combined with hypoxemia and hemorrhagic shock, we studied the status of peripheral redox systems, liver glycogen content, creatinine clearance, and systemic inflammation. Male Sprague-Dawley rats were subjected to hypoxemia and hemorrhagic shock insults (HH), penetrating ballistic-like brain injury (PBBI) alone, or PBBI followed by hypoxemia and hemorrhagic shock (PHH). Sham rats received craniotomy only. Biofluids and liver, kidney, and heart tissues were collected at 1 day, 2 days, 7 days, 14 days, and 28 days post-injury (DPI). Creatinine levels were measured in both serum and urine. Glutathione levels, glycogen content, and superoxide dismutase (SOD) and cytochrome C oxidase enzyme activities were quantified in the peripheral organs. Acute inflammation marker serum amyloid A-1 (SAA-1) level was quantified using western blot analysis. Urine to serum creatinine ratio in PHH group was significantly elevated on 7–28 DPI. Polytrauma induced a delayed disruption of the hepatic GSH/GSSG ratio, which resolved within 2 weeks post-injury. A modest decrease in kidney SOD activity was observed at 2 weeks after polytrauma. However, neither PBBI alone nor polytrauma changed the mitochondrial cytochrome C oxidase activity. Hepatic glycogen levels were reduced acutely following polytrauma. Acute inflammation marker SAA-1 showed a significant increase at early time-points following both systemic and brain injury. Overall, our findings demonstrate temporal cytological/tissue level damage to the peripheral organs due to combined PBBI and systemic injury.

## Introduction

Combat-associated traumatic brain injury (TBI) commonly occurs in combination with peripheral organ damage. However, the majority of TBI-polytrauma studies have focused primarily on the cerebral changes following combined injuries.^1–4^ Using a rat model of penetrating ballistic-like brain injury (PBBI) combined with hypoxemia and hemorrhagic shock (HH),^5,6^ the current study examines pathological changes in the peripheral organs. Further, to understand the role of systemic inflammation, serum amyloid A1 (SAA1) levels were measured.

Multiple systemic and local changes occur following TBI-polytrauma. The bioenergetics and redox status in peripheral organs are important outcome metrics for designing novel pharmacological interventions. Systemic hypoxemia and hypoperfusion reduce oxygen supply to the peripheral organs, leading to an energy deficit. Part of this deficit can be compensated for by the intracellular degradation of stored adenosine triphosphate (ATP) to adenosine diphosphate (ADP) and adenosine monophosphate (AMP).^7^ However, a persistent energy crisis will deplete ATP stores and lead to hepatic glycogenolysis.^8^ Therefore, hepatic glycogen content represents an indirect measurement of liver stress.

Another marker for metabolic stress is altered complex IV (cytochrome c oxidase) enzyme activity. Complex IV activity in major organ tissues, like heart and liver, is associated with bioenergetics imbalance and subsequent tissue stress.^9^ Increased oxidative stress is another important factor contributing to secondary injury. Superoxide dismutase (SOD) enzyme and glutathiones (GSH/GSSG) are the major reactive oxygen scavenging systems and perturbations in these systems indicate stress response at the tissue level. While alterations in brain SOD and GSH levels were detected in rodent models of TBI,^10–12^ no studies have examined the peripheral levels of these factors following either TBI alone or combined HH and TBI. We hypothesize that TBI, combined with systemic injury, results in metabolic abnormalities and oxidative stress in peripheral organs altering the endogenous antioxidant mechanisms.

TBI elicits both central and peripheral inflammatory responses. Multiple inflammatory factors are released into the bloodstream that can evoke inflammation in peripheral organs.^13^ An important marker of acute systemic inflammation is SAA1 protein; SAA1 is expressed in the liver and serves as a mediator for further inflammatory reactions.^14^ Serum levels of SAA1 have been found to be acutely upregulated in a mouse model of mild-to-moderate TBI.^13^ Although SAA1 expression is induced by cytokines, once it is released, SAA1 can further enhance expression of cytokines and chemokines, potentiating the inflammatory damage.^15^ To better understand the acute inflammatory response, we evaluated the temporal profile of serum SAA1 levels in the current study. Critically, our experiments will shed light on the systemic pathological aspect of polytrauma that has not been described previously.

## Methods

### Animals

All procedures involving animal use were reviewed and approved by the Institutional Animal Care and Use Committee of Walter Reed Army Institute of Research. Research (Protocol #WRAIR-13-PN-21S) was conducted in an Association for Assessment and Accreditation of Laboratory Animal Care International–accredited facility in compliance with the Animal Welfare Act and other federal statutes and regulations relating to animals and experiments involving animals, and adheres to principles stated in the *Guide for the Care and Use of Laboratory Animals,* NRC Publication, 2011 edition. Adult male Sprague-Dawley rats (280–320 g; Charles River Labs, Raleigh, VA) were used in these experiments. Animals were kept individually under a 12-h light/dark cycle, for a week for acclimatization prior to the experiment. Prior to surgery, each rat was maintained on a five-pellet (approximately 20–25 g) per day diet (Purina Mill Lad Diet: Prolab RMH 3000), and water was provided *ad libitum.* Food and water were given *ad libitum* after the surgical procedures. Rats were randomly assigned into one of four groups: sham, HH, PBBI, and PBBI combined with HH (PHH), with a sample size of 10 per group per time-point. Baseline measurements were done in the same rats (*n* = 50/group; 10 × 5 time-points) that were used for injuries.

### PBBI

In all animals, anesthesia was induced with 3% isoflurane, reduced to 2.5% for the initial surgeries, and maintained at 1.5% for the remaining procedures in a breathing air/oxygen mixture (fraction of inspired oxygen [FiO_2_] = 0.26). The right femoral artery and vein were cannulated for blood pressure monitoring and fluid resuscitation, respectively. In addition, the tail artery was cannulated for inducing hemorrhagic shock by withdrawing blood. All the animal surgery procedures were done in an aseptic manner. Care was taken to sanitize the PBBI probes in between multiple surgeries. The animal head was then fixed on a stereotaxic apparatus. Prior to all surgical incisions, lidocaine was injected subcutaneously near the surgical site.

Following a midline incision, the skull was exposed, the craniotomy location was marked on the cranium (+4.5 mm anterio-posterior, +2 mm medio-lateral from bregma) and a cranial window was created using a hand-held drill. PBBI was induced using a simulated ballistic injury device (Mitre Corp., McLean, VA) with a specially designed stainless steel probe (Popper & Sons Inc., Hyde Park, NY). The probe was mounted to a stereotaxic arm at an angle of 50° from the vertical axis and 25° counter-clockwise from the anterior-posterior axis. It was then manually inserted through the right frontal cortex via a cranial window to a distance of 12 mm (from dura). The elastic tubing on the probe was inflated by a rapid (< 40 msec) water pressure pulse, forming an elliptical balloon calibrated to 10% of the total rat brain volume to produce an intracerebral temporary cavity. The probe was then gently retracted and the cranial opening was sealed with sterile bone wax, and the incision was closed by wound clips.^16^ During the surgery procedure, blood pressure (at femoral artery using a research grade blood pressure transducer; Harvard Apparatus, Holliston, MA), rectal temperature, heart rate and breathing were continuously monitored and recorded using a data acquisition system (PowerLab, AD instruments, Colorado Springs, CO). Animals in the sham control and HH groups received craniotomy only (no probe insertion or balloon expansion).

### Polytrauma: combined PBBI, hypoxemia, and hemorrhagic shock

Hypoxemia (HX) was initiated 5 min after baseline recording or PBBI and maintained for 30 min in the HH or polytrauma (PHH) groups. HX was induced by replacing air with nitrogen in the inhalation gas mixture until the F_i_O_2_ reached 0.1 (monitored by respiratory gas analyzer). At the end of HX, blood gas was measured (ABL5, Radiometer, Copenhagen, Denmark) to ensure the partial pressure of arterial oxygen (P_a_O_2_) dropped below 40 mm Hg. Normoxia (F_i_O_2_ = 0.26) was restored by switching the nitrogen back to breathing air. Hemorrhagic shock (HS) was then induced by withdrawing blood via the tail arterial catheter using a withdrawal pump (Harvard Apparatus) at a constant rate of 0.25 mL/100 g/min to reduce mean arterial pressure (MAP) to 30–45 mm Hg. The hypotensive state was maintained for 30 min. Lactated Ringer's solution [LRS] Hospira, Lake Forest, IL) was given if MAP dropped below 30 mm Hg during the 30-min HS phase. At the end of the HS phase, LRS (three times the blood volume withdrawn) was infused at a rate of 0.9 mL/min (fluid resuscitation phase). After fluid resuscitation, all catheters were removed, and incisions were closed with sutures. Animals in the sham control group received the same procedures that the injury groups did, except for the injuries and fluid resuscitation.

### Food and water intake and urine output

One day prior to injury and at the specified end-points (1 day, 2 days, 7 days, 14 days, and 28 days post-injury), animals were placed in metabolic cages (Harvard Apparatus) in which food (grams of chow consumed over 6 h) and water intake, as well as urine output were recorded. All rats were placed in metabolic cages for about 6 h (started approximately at 08:30 and ended at 14:30). After 6 h was over, rats were removed from the metabolic cages and returned to their home cages. Urine, remaining water, and remaining rat chow were measured and recorded. If rats had not yet urinated after 6 h, they were tickled gently to induce urination. Urine was collected to a chilled collection cup placed on ice and centrifuged at 5000 rpm for 5 min at 4°C, to remove any undissolved material, and supernatant was stored at -80°C, until subsequent processing/analyses.

### Sample collection

For each time-point, separate cohorts were used. One day prior to injury, blood and urine samples were collected for baseline analysis. Baseline blood sample was collected through the tail artery prior to the injuries. At the specified end-points (1 day, 2 days, 7 days, 14 days, and 28 days post-injury), a terminal cardiac puncture was performed, and blood samples were collected for serum separation and subsequently, tissue samples from liver, kidney, and heart were collected and processed for various analyses.

### Creatinine assay

Urine creatinine was measured using creatinine assay kit (MAK080; Sigma-Aldrich Corp., St Louis, MO) according to the manufacturer's instructions. Briefly, samples were passed through 10 KDa cut off filter to sieve out unwanted proteins. The urine creatinine concentration was measured by a colorimetric assay at 530 nm using a microplate reader (Synergy MX; Biotek Instruments Inc., VT). Serum creatinine concentration was measured with a serum-specific assay (700460; Cayman Chemical, Ann Arbor, MI), according to the manufacturer's protocol. This kit uses Jaffe's reaction, where picric acid in an alkaline condition reacts with creatinine to give a concentration dependent color.

### Heart and liver mitochondrial cytochrome C oxidase assay

At the end of each experiment, animals were anesthetized with a mixture of 70 mg/kg ketamine and 6 mg/kg xylazine and euthanized by decapitation. Peripheral organs including heart, liver, and kidney were collected immediately for biochemical analysis. Mitochondria were isolated from the heart and liver tissue in ice-cold isolation buffer (215 mM mannitol, 75 mM sucrose, 0.1% bovine serum albumin, and 20 mM HEPES with/without 1 mM EGTA, pH 7.2). The mitochondrial protein concentration was determined using the Pierce™ BCA protein assay kit (Thermo Fisher Scientific, Waltham, MA). Complex IV activity was assessed using a kinetic assay (K287; BioVision Incorporated, Milpitas, CA). The cytochrome oxidase activity (Units/mg) was calculated using the equation ΔOD/time (Δt)÷ɛX protein (mg) where: ΔOD is the difference in OD at time t1 and time t2 and Δt is the difference in time (t1 - t2; in min). ε is the molar extinction coefficient of reduced cytochrome C at 550 nm (i.e., 7.04 mM^−1^cm^−1^). Protein is the concentration of sample (mg) used per reaction.

### Liver and kidney SOD activity assay

SOD activity in the liver and kidney tissues was assessed using a SOD determination kit (19160; Sigma-Aldrich Corp.). Tissue was sonicated in lysis buffer (0.25 M sucrose, 10 mM Tris-HCl pH 7.4, 1 mM EDTA) centrifuged, and the supernatant collected for the SOD assay. In the presence of a reducing superoxide anion, WST-1, a highly water-soluble tetrazolium salt, is reduced to a water-soluble WST-1 formazan dye. The rate of reduction with oxygen is linearly correlated with the xanthine oxidase activity, which is inhibited by SOD. Therefore, the 50% inhibition activity of SOD can be determined by colorimetric method. Since the absorbance at 440 nm is proportional to the amount of superoxide anion, the SOD activity, as an inhibition activity, could be quantified by measuring the decrease in the color development at 440 nm.

### Liver and kidney GSH/GSSG assays

Reduced (GSH) to oxidized (GSSG) glutathione ratio in the liver and kidney tissues was determined using a commercially available quantification kit (G257-10; Dojindo Molecular Technologies, Inc., Rockville, MD). A fixed amount of tissue (1 g) was homogenized in 1ml of 5% sulfosalicylic acid. Homogenate was centrifuged at 8000 g for 10 min and the supernatant was diluted with deionized water. Total glutathione (GSH and GSSG) and GSSG in the supernatant were measured using a microplate reader at 412 nm. The amount of GSH was determined by subtracting the amount of GSSG from the total glutathione.

### Hepatic glycogen

Hepatic glycogen level was measured using a commercially available assay kit (ab65620; Abcam, Cambridge, MA) per the manufacturer's instructions. The tissue sample was weighed and homogenized in distilled water using a tissue homogenizer. The homogenate was kept on a boiling water bath for 10 min and quick chilled on ice, followed by 20-min centrifugation at 17,000 g. Glycogen level in the supernatant was determined based on the hydrolysis of glycogen to glucose catalyzed by glucoamylase in the assay.

### Quantitative SAA1 Western blotting

SAA1 level in the blood serum was measured using an automated Western blotting system (Peggy Sue™; Protein Simple, San Jose, CA). Both baseline and post-injury serum samples (5 uL) were mixed with buffer containing SDS, dithiothreitol, and fluorescent molecular weight standards. Heat denatured samples were loaded onto a microplate along with stacking and separation matrices, blocking and wash buffers, antibody (Rabbit polyclonal -SAA1 from Origene, 1:50, and secondary antibody 1:1000 from Protein Simple) and detection reagents. The Simple Western System loads samples and reagents automatically into the capillaries and proteins are separated by size as they go through the stacking and separating matrices. Separated proteins are then immobilized in the capillaries using ultraviolet method and are then blocked and incubated with primary antibody. Detection was done using horseradish peroxidase–conjugated secondary antibodies and chemiluminescent substrate. Following detection, analysis and quantification was performed using Compass software. The SAA1 protein was quantitated based on the area under the curve of each band.

### Statistical analysis

The sample size is determined by power analysis (power = 0.8; alpha = 0.05) using G*Power 3 (Germany) based on the means and standard derivations of our preliminary data. Statistical analysis was performed using GraphPad Prism version 6.00 for Windows (GraphPad Software, La Jolla, CA) and SPSS v22 (IBM Corp, Armonk, NY). Shapiro-Wilk test was used to assess data normality (SPSS). Grubbs test was performed to identify outliers (GraphPad Prism) and we did not find outliers in any datasets presented in this manuscript. One-way analysis of variance (ANOVA), followed by Tukey's honestly significant difference or Newman-Keuls multiple comparison test, was used for data analysis at each time-point. The significance criterion was set at *p* < 0.05. Data are presented as the mean ± standard error of the mean (SEM).

## Results

### Body weight, food/water intake, and urine output

**FIG. 1.**
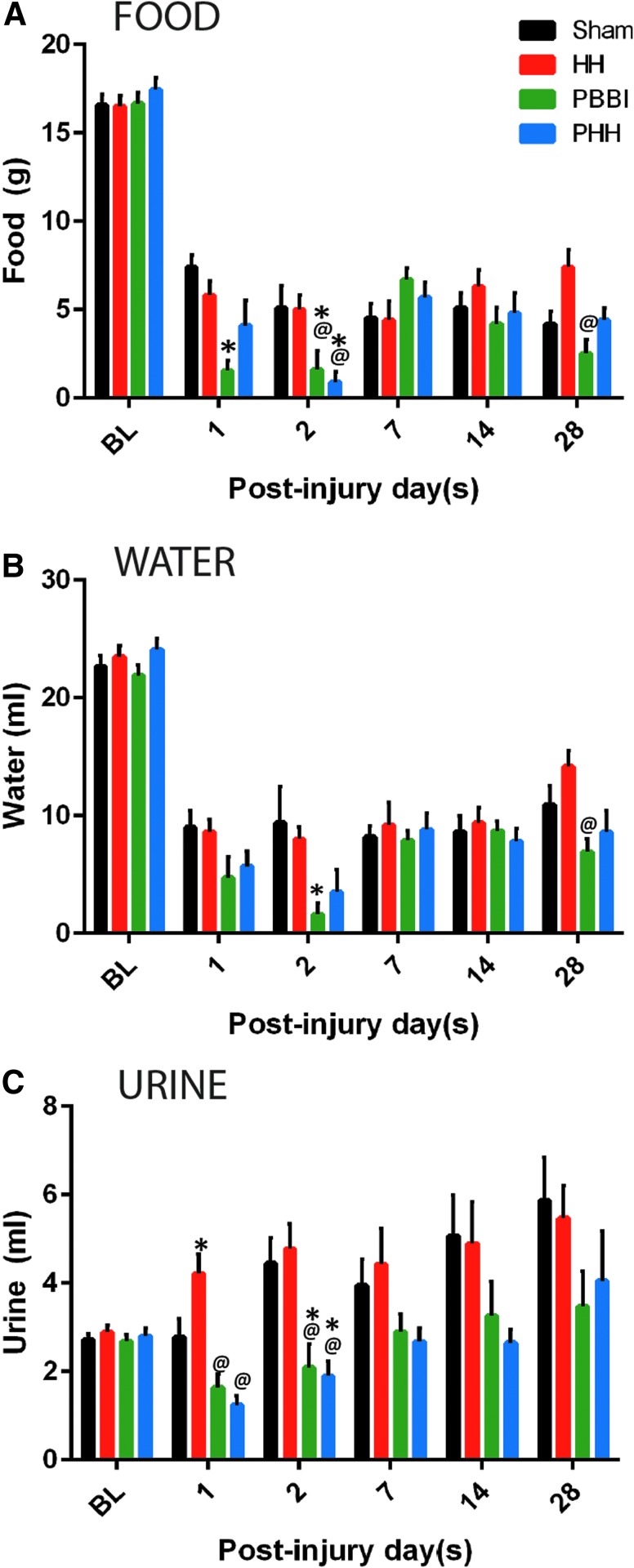
Food, water and urine levels following injury: Decreased food and water consumption were observed in rats following both polytrauma and penetrating ballistic-like brain injury (PBBI). Temporal changes in **(A)** food consumption, **(B)** water intake, and **(C)** urine output. Baseline (BL) data is from all 50 rats (10 rats from each time-point) per group and for the post-injury set it is *n* = 10 per group per time-point; *p* values for between-group analysis of variance was <0.008 for food, <0.019 for water and <0.001 for urine. *p < 0.05 vs. sham group; @*p* < 0.05 vs. hypoxemia and hemorrhagic shock (HH) group.

Compared with sham group, body weight showed a slight, non-significant reduction in both PBBI and PHH groups at 24 h, 48 h, and 7 days (Supplementary Fig. S1). Compared with the pre-injury intake, food and water consumption decreased drastically following injury in all of the injury groups, as well as in the sham control group (Fig. 1A, 1B). Although food and water intake improved over time, it remained lower than baseline at all time-points. Compared with the sham and HH animals, the food and water intake were significantly lower in the PBBI and PHH groups at acute time-points. Both PBBI and PHH induced a significant decrease in urine output compared with shams at 2 days post-injury (*p* = 0.029 for PBBI and *p* = 0.011 for PHH; Fig. 1C). At the later time-points, urine output was modestly, albeit not significantly, decreased in the PBBI and PHH groups.

### Creatinine excretion

**FIG. 2.**
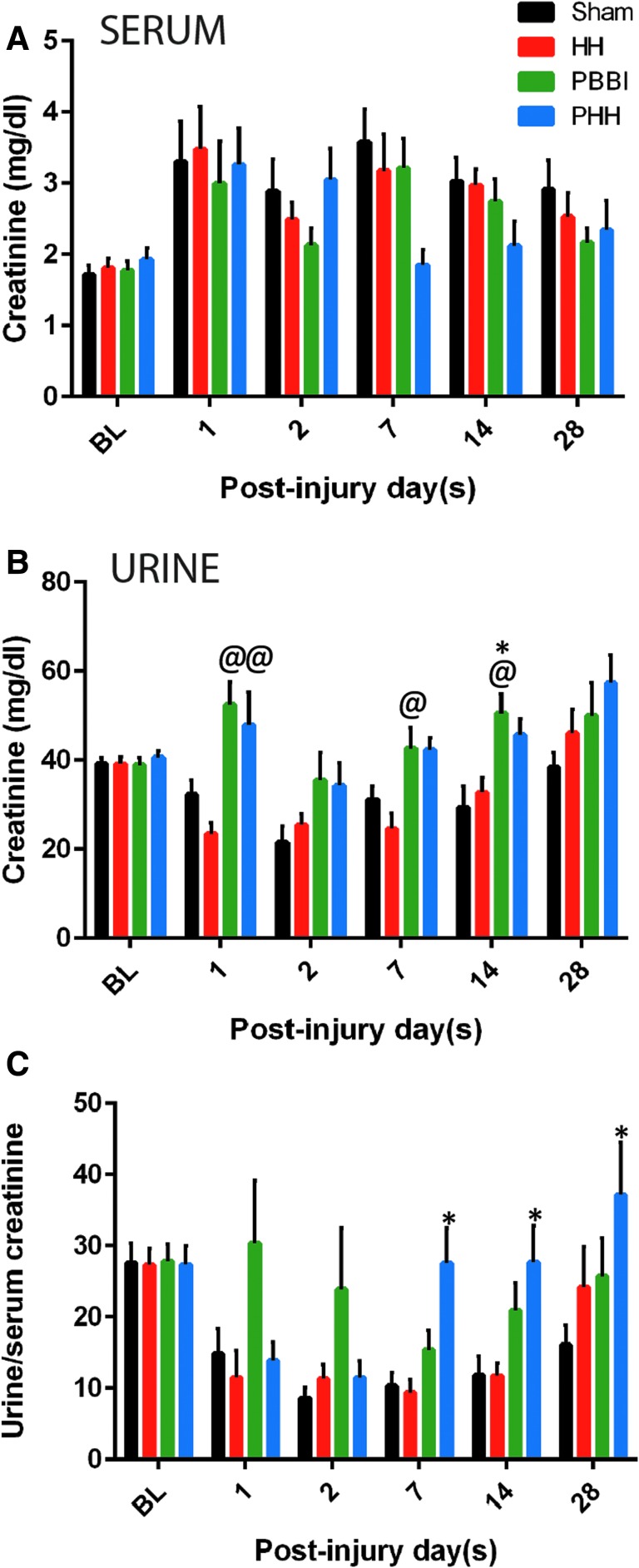
Creatinine levels following injury: Temporal changes in **(A)** serum creatinine, **(B)** urine creatinine, and **(C)** urine to serum creatinine ratio, showing increased creatinine clearance following polytrauma. Urine and serum creatinine values were assessed separately and normalized to the sample volume. Data represented as mean ± standard error of the mean. Baseline (BL) data is from all 50 rats (10 rats from each time-point) per group and for the post-injury set it is *n* = 10 per group per time-point; *p* value for between-group analysis of variance was <0.020 for urine creatinine. *p < 0.05 vs. sham control group. @*p* < 0.05 vs. hypoxemia and hemorrhagic shock (HH) group.

Urine and serum creatinine values were quantified separately and normalized to the volume. No significant differences in serum creatinine levels were detected between groups at any time-points. However, PHH animals showed a trend towards lower serum creatinine at 7 days post-injury (*p* = 0.099 vs. sham) compared with the other groups (Fig. 2A). Urine creatinine levels increased following PBBI or PHH at all time-points except 28 days post-injury (Fig. 2B). Interestingly, urine to serum creatinine ratio in the PHH group increased significantly starting at 1 week (*p* = 0.002 vs. sham) and remained high at 28 days post-injury (Fig. 2C).

### Cytochrome C and SOD enzyme activities

**FIG. 3.**
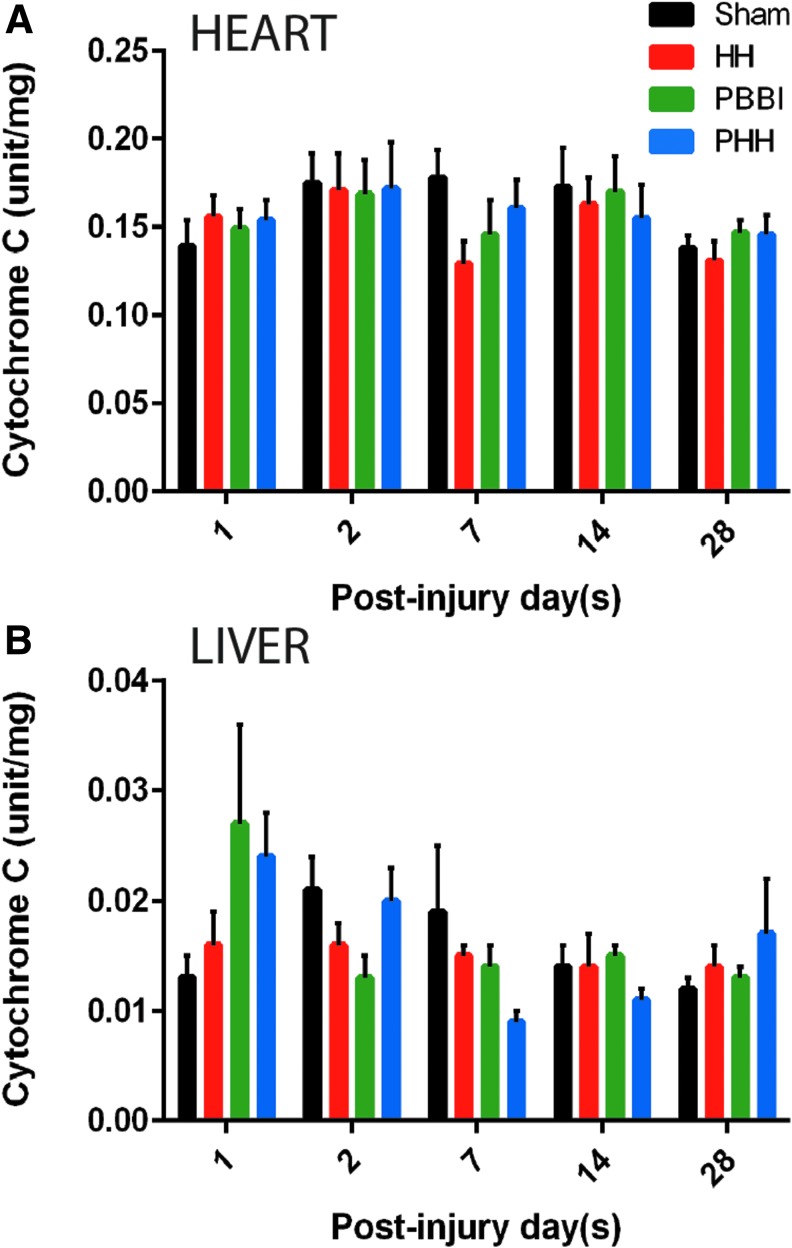
Cytochrome C oxidase activity in peripheral organs following injury: No significant change in mitochondrial cytochrome C oxidase activity was observed in either liver or heart tissue in any of the three groups compared with sham. Temporal changes in mitochondrial cytochrome C activity in **(A)** heart and **(B)** liver tissue. Values are expressed as mean ± standard error of the mean. *n* = 10 per group per time-point.

**FIG. 4.**
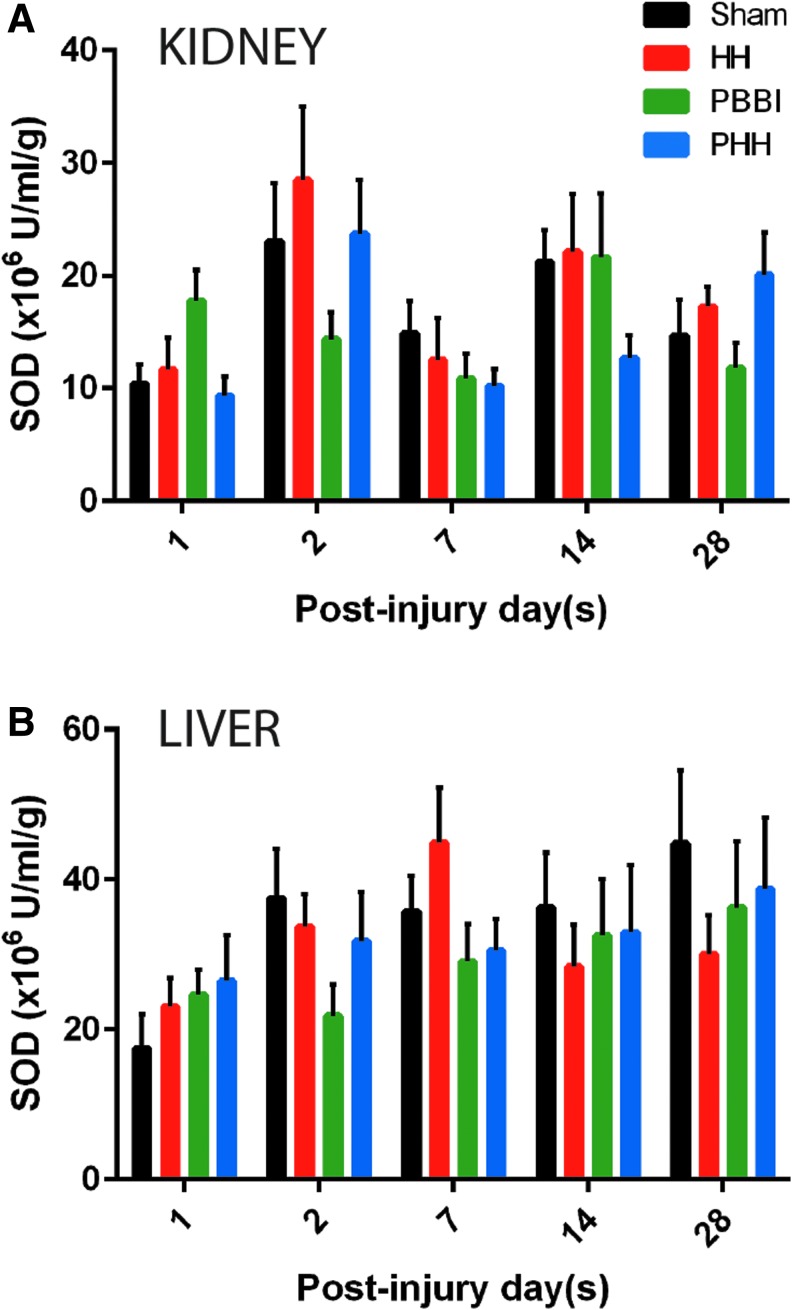
Super oxide dismutase activity following injury: No significant change in super oxide dismutase (SOD) activity was observed in either kidney or liver tissue in any of the three injury groups compared with sham. Temporal changes in SOD activity in **(A)** kidney and **(B)** liver tissue. *n* = 10 per group per time-point. Data expressed as mean ± standard error of the mean.

The mitochondrial cytochrome C activity in the liver and heart did not show any significant changes following injury compared with sham controls (Fig. 3). A trend toward a reduction of heart cytochrome C was detected in the HH and PBBI groups on Day 7 post-injury (*p* = 0.182; Fig. 3A). In contrast, there was an early increase in liver cytochrome C in the PBBI and PHH groups, although not statistically significant from the sham control group (Fig. 3B). Total SOD activity was measured in both liver and kidney tissue. No significant changes were observed in the SOD activity in either of these organs (Fig. 4A, 4B). There was a trend toward reduced SOD activity in the PHH group at 1 week post-injury in the kidney (*p* = 0.395; Fig. 4A). Overall, kidney SOD activity was found to be of lower magnitude, compared with the liver SOD activity.

### GSH/GSSG ratio and hepatic glycogen content

**FIG. 5.**
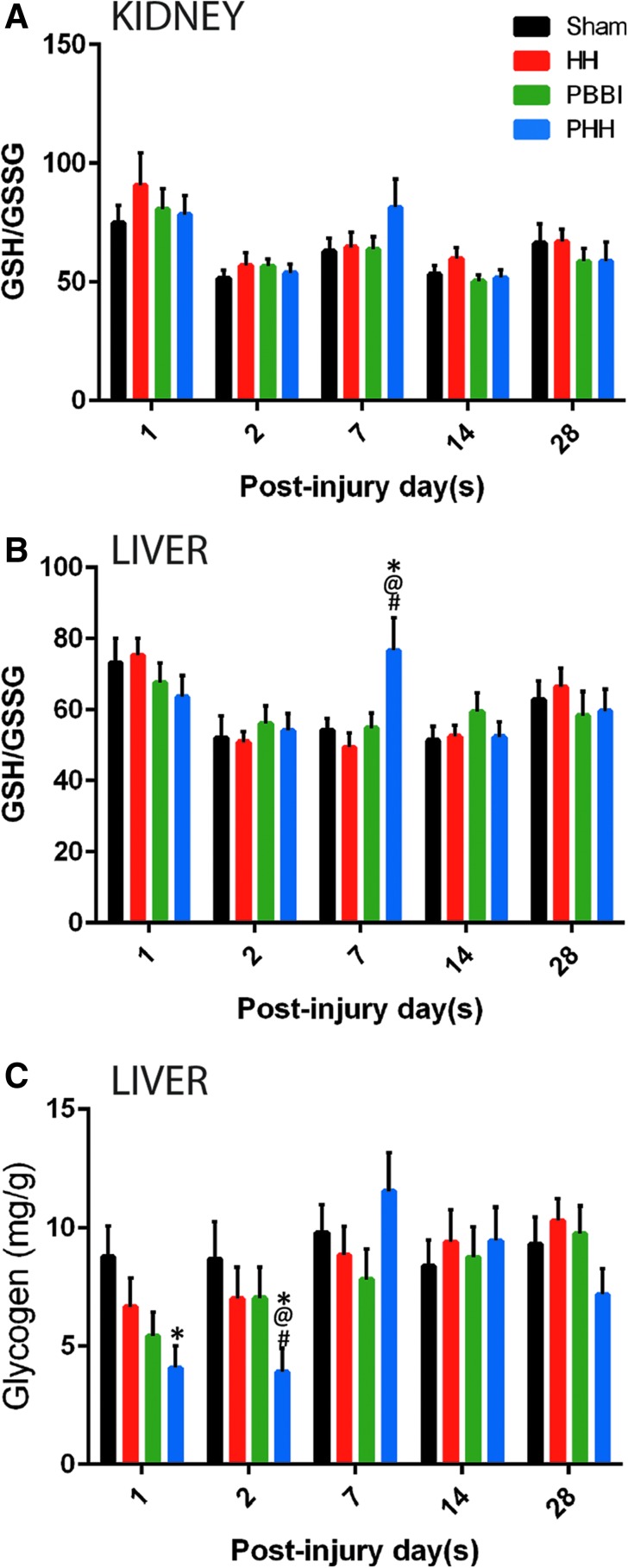
Systemic glutathione levels following injury: Polytrauma induced a significant increase in the ratio of reduced glutathione (GSH) to oxidized glutathione (GSSG) in the liver. Temporal changes in GSH/GSSG in **(A)** kidney and **(B)** liver following hypoxemia and hemorrhagic shock (HH), polytrauma or penetrating ballistic-like brain injury (PBBI) or sham procedures. **(C)** Temporal changes in liver glycogen content following injury. Polytrauma induced an acute reduction in hepatic glycogen content. Values are presented as mean ± standard error of the mean. *n* = 10 per group per time-point; *p* values for between-group analysis of variance was <0.027 for liver glutathione and <0.048 for hepatic glycogen. *p < 0.05 vs. sham control group; @*p* < 0.05 vs. HH group; #*p* < 0.05 vs. PBBI group.

PHH induced an increase in GSH/GSSG ratio in both liver and kidney at 1 week post-injury (Fig. 5A, 5B) that returned to sham levels by 2 weeks post-injury. Although the ratio in both organs exhibited the same trend, the increase was only significant in the liver (*p* = 0.006 vs. sham). For all the other groups, the GSH/GSSG ratio dropped at 2 days post-injury and remained low throughout the study duration. Hepatic glycogen content in the PHH group was significantly lower than the sham control group at 1 day (*p* = 0.024) and 2 days post-injury (*p* = 0.041; Fig. 5C). It returned to the level that was comparable to the other groups by 1 week post-injury. No significant differences were observed between the sham, HH, and PBBI groups at any of the time-points.

### Serum SAA1 levels

**FIG. 6.**
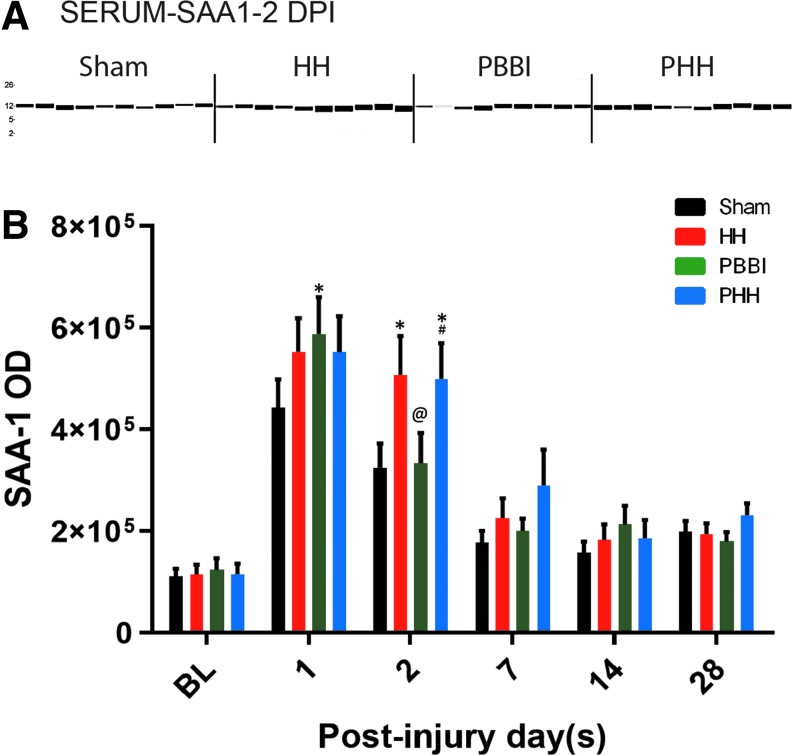
Serum amyloid A1 (SAA1) levels following injury: SAA1 expression levels in the serum samples collected pre-injury and also following hypoxemia and hemorrhagic shock (HH), penetrating ballistic-like brain injury (PBBI) and polytrauma. **(A)** Representative western blot image from 2-day time-point. (B) Quantification of SAA1 levels across multiple time-points. Baseline (BL) data is from 30 rats (six rats from each time-point) per group and for the post-injury set it is *n* = 9–10 per group per time-point; Values are presented as mean ± standard error of the mean. *p* value for between-group analysis of variance was <0.0089. *p < 0.05 vs. sham control group; #*p* < 0.05 vs. PBBI group. DPI, days post-injury.

Baseline SAA1 levels were comparable among the four groups. The serum SAA1 level peaked on Day 1 post-injury in all groups (Fig. 6). In particular, all injury groups had a higher SAA1 level than the sham control group, although the increase was significant only in the PBBI group (*p* = 0.035 vs. sham). However, this increase was transient as the expression in the PBBI group went back to sham control levels by Day 2 post-injury. On the other hand, HH and PHH groups showed a more robust increase in SAA1 levels (*p* = 0.0091 vs. sham for HH and *p* = 0.0127 vs. sham for PHH on Day 2 post-injury). Interestingly, the serum SAA1 levels in all groups showed a reduction and returned to baseline levels by 1 week post-injury, and remained low at 14 and 28 days post-injury.

## Discussion

In the present study, we examined the temporal alterations in peripheral organs in a rat model of combined PBBI and systemic insults. Specifically, general health indicators, as well as specific systemic parameters for inflammation and organ function were measured at acute, subacute and chronic time-points.

Acute reduction in food and water intake was observed following PBBI and polytrauma compared with the sham group. In addition, both injury groups showed slight reduction in body weight up to 7 days post-injury. This was not unexpected as brain injury can cause anorexia and weight loss in patients^17,18^ and in animal models.^19^ An interesting observation in our study is that voluntary food and water intake were reduced in all groups including shams compared with pre-injury level. This non-specific response may be in part due to the stress response to the surgery procedure. Sham rats received only craniotomy, which appears to be sufficient to produce anorexic response. Intriguingly, none of the groups resumed their pre-injury food and water consumption, indicative of the chronic stress response to surgical procedures. Supporting our notion, it was previously reported that exposure of rats to physical or psychological stressors caused changes in their food intake that persisted for days after initial exposure to the stressors.^20^ This lower consumption of food and water also may be due to hormonal disturbances following injury.^21^ Although we cannot explain it with the current study design, another plausible reason for reduced food and water intake is low gastric emptying. Reduced gastric emptying is reported following TBI due to the elevated intracranial pressure and subsequent depression of vagus nerve activity after TBI.^22–24^ In our previous study, we measured intracranial pressure (ICP) continuously for ∼6 h after PBBI (unpublished data) and ICP remained elevated. In another study, significant increase in brain water content, indicative of brain edema, was detected at 4 h to 7 days after PBBI.^25^ Therefore, it is likely that ICP was elevated in this cohort as well that may have interfered with gastric emptying.

Urine output in the PBBI and polytrauma groups remained low throughout the study period. This could be partly explained by their low water intake. Additionally, sympathetic storming after TBI may lead to urine retention.^26^ Low water intake and urine output can alter plasma osmolarity, that was however not measured in the current study. We detected an increased urine to serum creatinine ratio indicating augmented renal clearance (ARC) after PBBI-polytrauma. Acute renal dysfunction has been observed in patients with severe TBI, as indicated by persistent high creatinine levels in the serum.^27^ Notably, Udy and colleagues reported a high incidence (85%) of augmented creatinine clearance in a group of young TBI patients, which may be associated with the active upkeep of cerebral perfusion pressure.^28^ ARC is usually observed in critically ill patients and an elevated ARC can interfere with renally cleared drugs leading to treatment failure.^29^ It is therefore important to measure ARC while designing treatment options for TBI combined with systemic injuries.

Cytochrome C is a mitochondrial protein that is essential for aerobic energy production; therefore, we measured the cytochrome C activity in both heart and liver mitochondria. Cytochrome C oxidase activity did not change following PBBI or polytrauma in heart mitochondria; however, liver mitochondria showed a modest increase followed by a reduction. This indicates that the liver mitochondria are more sensitive to the injury(s) in our model, compared with the heart mitochondria. In fact, metabolic alterations in the liver are believed to be associated with brain injury.^30^ Moreover, liver weight and hepatic protein content are significantly reduced following fluid percussion injury in rats.^19^ Similar findings were observed in a clinical study that noted incidences of hepatic and renal dysfunction after severe brain injury were 8% and 7% respectively.^31^ TBI and polytrauma are known to induce the liver stress markers alanine amino transferase and aspartate amino transferase.^32,33^ Although there was a slight decrease in liver cytochrome C activity at 1 week post-injury in the PHH group, this does not necessarily imply an impaired energy homeostasis. Critically, hepatic glycogen in the PHH group at this particular time-point was comparable to levels in sham control rats, whereas it was significantly lower than all groups at 2 days post-injury. The elevated level of glucocorticoids after severe brain injury and its effects on hepatic gluconeogenesis and glycogenesis might account for these changes.^34–36^

In our study, TBI alone did not change the hepatic GSH levels. Interestingly, additional hypoxemic and hypotensive insults after TBI increased the reduced glutathione levels after 1 week post-injury, indicating delayed oxidative stress in the liver. This injury response might imply a mechanism to restore homeostasis within the liver cells. Few studies have reported on hepatic oxidative stress, inflammation, and altered hepatic redox status following brain injury.^37,38^ In the rat model of fluid percussion injury, the following changes were detected during the acute phase of the injury: increased hepatic inflammation (cyclooxygenase-2, inducible nitric oxide synthase, tumor necrosis factor [TNF]-α, and interleukin [IL]-6), oxidative stress indicated by the reduced glutathione level; altered hepatic redox status; mitochondrial dysfunction^37^; and reduced hepatic transcription factor expression.^38^ Similarly, clinical studies showed that the plasma level of GSH at 1 day post-injury was found to be higher in mild TBI patients compared with that of the control group.^39^ In another study, the GSH level in the cerebrospinal fluid was elevated at 1 day post-injury but returned to the control level at 2 days post-injury.^40^ Even though, both pre-clinical and clinical TBI studies reported altered glutathione levels in the periphery, in the current study, PBBI alone did not alter GSH levels in the liver or kidney. It is possible that the temporal course of GSH changes in penetrating injury model is different from other TBI models and we may have missed capturing that in the current study.

We also examined the enzyme activity for SOD in both liver and kidney. Antioxidant enzyme SOD reduces superoxide molecules to hydrogen peroxide that is subsequently detoxified by a catalase. Although the total SOD activity in liver was higher than that in the kidney, it did not appear to be affected by the TBI itself or the combined injuries.

Our data showed that TBI alone induced a transient increase in serum SAA1 levels. A more robust increase of SAA1 levels was detected following HH or PHH. Given the fact that the liver is the major source of SAA1,^15^ the hepatic stress induced by HH and PHH may have contributed to this effect.^13^ Interestingly, SAA1 levels increased in all groups at acute time-points, suggesting SAA1 is highly sensitive, and capable of detecting mild trauma caused by the sham procedures (cannulations, scalp incision, and craniotomy). While the function of SAA1 is not fully understood, it is a strong pro-inflammatory mediator involved in the production of many cytokines such as IL-1A, IL-1B, IL-8, IL-6, and TNFα.^41,42^ It has been suggested that SAA1 can serve as a biomarker for TBI.^32,33^ SAA1 also plays an important role in systemic acute phase response,^43^ in which circulating inflammatory cells become accumulated in peripheral organs like the liver, kidney, lungs, and heart, and can lead to inflammation in these organs.^44–47^ In particular, the liver is a major organ that induces and regulates these kinds of responses.^47,48^ Following TBI, activated inflammatory cells are not restricted to the brain parenchyma, but neutrophil and leukocyte recruitment also occurs in the peripheral system.^49^

Although we characterized systemic responses following polytrauma, there are a few limitations to the current study. We could not measure changes in the lung tissue as this was not collected during sampling. Another major limitation is the lack of histological examination. We designed the study for biochemical experiments and another cohort was necessary to perform histology. Brain tissue from the current study is being used for another project that will explore both histological and biochemical changes in the brain following polytrauma.

## Conclusion

Collectively, our study profiled the temporal changes of various systemic parameters following PBBI with or without additional hypoxemic and hypotensive insults. Our results reflect temporal cytological/tissue level damage to the peripheral organs due to combined PBBI and systemic injury. Although the majority of the systemic parameters were unaltered after PBBI or PBBI-polytrauma, we identified changes in the peripheral redox systems, such as hepatic glutathione, which indicates oxidative stress. Increased renal creatinine clearance was also observed after combined insults, indicating the effect of PBBI and polytrauma on kidney function. Our findings suggest that when combined with TBI, systemic injury can affect peripheral organ function, and these parameters need to be considered when designing therapeutic treatments for TBI-polytrauma.

## Supplementary Material

Supplemental data

## References

[1] Dennis A.M., M.L. Haselkorn V.A. Vagni R.H. Garman K. J anesko-Feldman H. B ayir R.S. Clark L.W. Jenkins C.E., and Dixon P.M Kochanek. (2009). Hemorrhagic shock after experimental traumatic brain injury in mice: effect on neuronal death. J. Neurotrauma 26, 889–899 18781889 10.1089/neu.2008.0512 PMC2694227

[2] Vogt N., C. Herden E. R oeb M. R oderfeld D. E schbach T. S teinfeldt H. W ulf S. R uchholtz E. Uhl, andScholler K. (2018). Cerebral alterations following experimental multiple trauma and hemorrhagic shock. Shock 49, 164–173 28682946 10.1097/SHK.0000000000000943

[3] Proctor J.L., D. Scutella Y. P an J. V aughan R.E. Rosenthal A., and Puche G Fiskum. (2015). Hyperoxic resuscitation improves survival but worsens neurologic outcome in a rat polytrauma model of traumatic brain injury plus hemorrhagic shock. J. Trauma Acute Care Surg. 79, S101–S109 26406421 10.1097/TA.0000000000000742

[4] Foley L.M., A.M. Iqbal O'Meara S.R. Wisniewski T.K. Hitchens J.A. Melick C. Ho, Jenkins L.W. , and Kochanek P.M. (2013). MRI assessment of cerebral blood flow after experimental traumatic brain injury combined with hemorrhagic shock in mice. J. Cereb. Blood Flow Metab. 33, 129–136 23072750 10.1038/jcbfm.2012.145 PMC3597358

[5] Leung L.Y., Y. Deng-Bryant D., andShear F Tortella. (2015). Combined hypoxemic and hypotensive insults altered physiological responses and neurofunction in a severity-dependent manner following penetrating ballistic-like brain injury in rats. J. Trauma Acute Care Surg. 79, S130–S138 26406425 10.1097/TA.0000000000000785

[6] Leung L.Y., Y. Deng-Bryant K. C ardiff M. W inter F., and Tortella D Shear. (2016). Neurochemical changes following combined hypoxemia and hemorrhagic shock in a rat model of penetrating ballistic-like brain injury: a microdialysis study. J. Trauma Acute Care Surg. 81, 860–867 27769083 10.1097/TA.0000000000001206

[7] Keel M. and Trentz O. (2005). Pathophysiology of polytrauma. Injury. 36, 691–709 15910820 10.1016/j.injury.2004.12.037

[8] Petersen M.C., D.F. Vatner, and Shulman G.I. (2017). Regulation of hepatic glucose metabolism in health and disease. Nat. Rev. Endocrinol. 13, 572–587 28731034 10.1038/nrendo.2017.80 PMC5777172

[9] Jaeschke H., M.R. McGill, andRamachandran A. (2012). Oxidant stress, mitochondria, and cell death mechanisms in drug-induced liver injury: lessons learned from acetaminophen hepatotoxicity. Drug Metab. Rev. 44, 88–106 22229890 10.3109/03602532.2011.602688 PMC5319847

[10] Bayir H., V.E. Kagan R.S.B. Clark K. J anesko-Feldman R. R afikov Z.T. Huang X.J. Zhang V. V agni T.R. Billiar, andKochanek P.M. (2007). Neuronal NOS-mediated nitration and inactivation of manganese superoxide dismutase in brain after experimental and human brain injury. J. Neurochem. 101, 168–181 17394464 10.1111/j.1471-4159.2006.04353.x

[11] Rodriguez-Rodriguez A., J.J. Egea-Guerrero F. Murillo-Cabezas, andCarrillo-Vico A (2014). Oxidative stress in traumatic brain injury. Curr. Med. Chem. 21, 1201–1211 24350853 10.2174/0929867321666131217153310

[12] Ansari M.A., K.N. Roberts, andScheff S.W. (2008). A time course of contusion-induced oxidative stress and synaptic proteins in cortex in a rat model of TBI. J. Neurotrauma 25, 513–526 18533843 10.1089/neu.2007.0451

[13] Villapol S., D. Kryndushkin M.G. Balarezo A.M. Campbell J.M. Saavedra F.P. Shewmaker, andSymes A.J (2015). Hepatic expression of serum amyloid A1 is induced by traumatic brain injury and modulated by telmisartan. Am. J. Pathol. 185, 2641–2652 26435412 10.1016/j.ajpath.2015.06.016 PMC4607758

[14] Urieli-Shoval S., R.P. Linke, andMatzner Y. (2000). Expression and function of serum amyloid A, a major acute-phase protein, in normal and disease states. Curr. Opin. Hematol. 7, 64–69 10608507 10.1097/00062752-200001000-00012

[15] Lindhorst E., D. Young W. B agshaw M. Hyland, andKisilevsky R. (1997). Acute inflammation, acute phase serum amyloid A and cholesterol metabolism in the mouse. Biochim. Biophys. Acta 1339, 143–154 9165109 10.1016/s0167-4838(96)00227-0

[16] Williams A.J., J.A. Hartings X.C. Lu M.L. Rolli J.R. Dave, andTortella F.C. (2005). Characterization of a new rat model of penetrating ballistic brain injury. J. Neurotrauma 22, 313–331 15716636 10.1089/neu.2005.22.313

[17] Lewin J. andSumners D (1992). Anorexia due to brain injury. Brain Inj. 6, 199–201 1571725 10.3109/02699059209029660

[18] Trummer M., S. Eustacchio F. U nger M. Tillich, andFlaschka G. (2002). Right hemispheric frontal lesions as a cause for anorexia nervosa report of three cases. Acta Neurochir. (Wien). 144, 797–801 12181689 10.1007/s00701-002-0934-5

[19] Moinard C., N. Neveux N. R oyo C. G enthon C. M archand-Verrecchia M. Plotkine, andCynober L (2005). Characterization of the alteration of nutritional state in brain injury induced by fluid percussion in rats. Intensive Care Med. 31, 281–288 15703899 10.1007/s00134-004-2489-9

[20] Valles A., O. Marti A. Garcia, andArmario A. (2000). Single exposure to stressors causes long-lasting, stress-dependent reduction of food intake in rats. Am. J. Physiol. Regul. Integr. Comp. Physiol. 279, R1138–1144 10956276 10.1152/ajpregu.2000.279.3.R1138

[21] Stengel A. andTache Y (2009). Neuroendocrine control of the gut during stress: corticotropin-releasing factor signaling pathways in the spotlight. Annu. Rev. Physiol. 71, 219–239 18928406 10.1146/annurev.physiol.010908.163221 PMC2714186

[22] Brinson R.R. andKolts B.E (1987). Hypoalbuminemia as an indicator of diarrheal incidence in critically ill patients. Crit. Care Med. 15, 506–509 3105959 10.1097/00003246-198705000-00011

[23] Guenter P.A., R.G. Settle S. P erlmutter P.L. Marino G.A. DeSimone, and Rolandelli R.H. (1991). Tube feeding-related diarrhea in acutely Ill patients. JPEN J. Parenter. Enteral Nutr. 15, 277–280 1650854 10.1177/0148607191015003277

[24] Tan M., J.C. Zhu, andYin H.H. (2011). Enteral nutrition in patients with severe traumatic brain injury: reasons for intolerance and medical management. Br. J. Neurosurg. 25, 2–8 21323401 10.3109/02688697.2010.522745

[25] Shear D.A., X.C. Lu R. P edersen G. W ei Z. C hen A. D avis C. Y ao J. Dave, andTortella F.C. (2011). Severity profile of penetrating ballistic-like brain injury on neurofunctional outcome, blood-brain barrier permeability, and brain edema formation. J. Neurotrauma 28, 2185–2195 21644814 10.1089/neu.2011.1916

[26] Feng Y., X. Zheng, andFang Z (2015). Treatment progress of paroxysmal sympathetic hyperactivity after acquired brain injury. Pediatr. Neurosurg. 50, 301–309 26352612 10.1159/000439282

[27] Li N., W.G. Zhao, andZhang W.F (2011). Acute kidney injury in patients with severe traumatic brain injury: implementation of the acute kidney injury network stage system. Neurocrit. Care 14, 377–381 21298359 10.1007/s12028-011-9511-1

[28] Udy A.,R. Boots S. S enthuran J. S tuart R. D eans M. Lassig-Smith, andLipman J (2010). Augmented creatinine clearance in traumatic brain injury. Anesth. Analg. 111, 1505–1510 21048095 10.1213/ANE.0b013e3181f7107d

[29] Hobbs A.L.V., K.M. Shea K.M. Roberts, andDaley M.J (2015). Implications of augmented renal clearance on drug dosing in critically ill patients: a focus on antibiotics. Pharmacotherapy 35, 1063–1075 26598098 10.1002/phar.1653

[30] Moinard C., S. Gupta V. B esson B. M orio C. M archand-Leroux J.C. Chaumeil L. Cynober, andCharrueau C (2008). Evidence for impairment of hepatic energy homeostasis in head-injured rat. J. Neurotrauma 25, 124–129 18260795 10.1089/neu.2007.0391

[31] Zygun D.A., J.B. Kortbeek G.H. Fick K.B. Laupland, andDoig C.J (2005). Non-neurologic organ dysfunction in severe traumatic brain injury. Crit. Care Med. 33, 654–660 15753760 10.1097/01.ccm.0000155911.01844.54

[32] Sanfilippo F., T. Veenith C. S antonocito C.S. Vrettou, andMatta B.F (2014). Liver function test abnormalities after traumatic brain injury: is hepato-biliary ultrasound a sensitive diagnostic tool? Br. J. Anaesth. 112, 298–303 24067331 10.1093/bja/aet305

[33] Fox A., J.B. Sanderlin S. M cNamee J.S. Bajaj W. Carne, andCifu D.X (2014). Elevated liver enzymes following polytraumatic injury. J. Rehabil. Res. Dev. 51, 869–874 25479083 10.1682/JRRD.2013.10.0233

[34] Feldman Z., C.F. Contant R. P ahwa J.C. Goodman C.S. Robertson R.K. Narayan, andGrossman R.G (1993). The relationship between hormonal mediators and systemic hypermetabolism after severe head injury. J. Trauma 34, 806–816 8315675 10.1097/00005373-199306000-00010

[35] Hill A.G. andHill G.L (1998). Metabolic response to severe injury. Br. J. Surg. 85, 884–890 9692557 10.1046/j.1365-2168.1998.00779.x

[36] Singer M., V. De Santis D. Vitale, andJeffcoate W (2004). Multiorgan failure is an adaptive, endocrine-mediated, metabolic response to overwhelming systemic inflammation. Lancet 364, 545–548 15302200 10.1016/S0140-6736(04)16815-3

[37] de Castro M.R.T., A.P.O. Ferreira G.L. Busanello L.R.H. da Silva M.E.P. da Silveira Junior F.D.S. Fiorin G. A rrifano M.E. Crespo-Lopez R.P. Barcelos M.J. Cuevas G. B resciani J. G onzalez-Gallego M.R. Fighera, andRoyes L.F.F (2017). Previous physical exercise alters the hepatic profile of oxidative-inflammatory status and limits the secondary brain damage induced by severe traumatic brain injury in rats. J. Physiol. 595, 6023–6044 28726269 10.1113/JP273933 PMC5577552

[38] Nizamutdinov D., S. DeMorrow M. M cMillin J. K ain S. M ukherjee S. Z eitouni G. F rampton P.C. Bricker J. Hurst, andShapiro L.A (2017). Hepatic alterations are accompanied by changes to bile acid transporter-expressing neurons in the hypothalamus after traumatic brain injury. Sci. Rep. 7, 40112 28106051 10.1038/srep40112 PMC5247752

[39] Wang H.C., Y.J. Lin F.Y. Shih H.W. Chang Y.J. Su B.C. Cheng C.M. Su N.W. Tsai Y.T. Chang A.L. Kwan, andLu C.H (2016). The role of serial oxidative stress levels in acute traumatic brain injury and as predictors of outcome. World Neurosurg. 87, 463–470 26481337 10.1016/j.wneu.2015.10.010

[40] Bayir H., V.E. Kagan Y.Y. Tyurina V. T yurin R.A. Ruppel P.D. Adelson S.H. Graham K. J anesko R.S. Clark, andKochanek P.M (2002). Assessment of antioxidant reserves and oxidative stress in cerebrospinal fluid after severe traumatic brain injury in infants and children. Pediatr. Res. 51, 571–578 11978879 10.1203/00006450-200205000-00005

[41] Yu Y., J. Liu S.Q. Li L. Peng, andYe R.D (2014). Serum amyloid a differentially activates microglia and astrocytes via the PI3K pathway. J. Alzheimers Dis. 38, 133–44 23948927 10.3233/JAD-130818

[42] Villapol S. (2016). Consequences of hepatic damage after traumatic brain injury: current outlook and potential therapeutic targets. Neural Regen. Res. 11, 226–227 27073366 10.4103/1673-5374.177720 PMC4810977

[43] Jensen L.E. andWhitehead A.S (1998). Regulation of serum amyloid A protein expression during the acute-phase response. Biochem. J. 334 (Pt 3), 489–503 10.1042/bj3340489 PMC1219714 9729453

[44] Campbell S.J., P.M. Hughes J.P. Iredale D.C. Wilcockson S. W aters F. D ocagne V.H. Perry, andAnthony D.C (2003). CINC-1 is an acute-phase protein induced by focal brain injury causing leukocyte mobilization and liver injury. FASEB J. 17, 1168–1170 12709409 10.1096/fj.02-0757fje

[45] Campbell S.J., V.H. Perry F.J. Pitossi A.G. Butchart M. C hertoff S. W aters R. Dempster, andAnthony D.C (2005). Central nervous system injury triggers hepatic CC and CXC chemokine expression that is associated with leukocyte mobilization and recruitment to both the central nervous system and the liver. Am. J. Pathol. 166, 1487–1497 15855648 10.1016/S0002-9440(10)62365-6 PMC1606402

[46] Campbell S.J., R.M. Deacon Y. J iang C. F errari F.J. Pitossi, andAnthony D.C. (2007). Overexpression of IL-1beta by adenoviral-mediated gene transfer in the rat brain causes a prolonged hepatic chemokine response, axonal injury and the suppression of spontaneous behaviour. Neurobiol. Dis. 27, 151–163 17580116 10.1016/j.nbd.2007.04.013

[47] Campbell S.J., I. Zahid P. L osey S. L aw Y. J iang M. B ilgen N. van Rooijen D. M orsali A.E. Davis, andAnthony D.C (2008). Liver Kupffer cells control the magnitude of the inflammatory response in the injured brain and spinal cord. Neuropharmacology 55, 780–787 18674548 10.1016/j.neuropharm.2008.06.074

[48] Baumann H. andGauldie J (1994). The Acute-Phase Response. Immunol. Today 15, 74–80 7512342 10.1016/0167-5699(94)90137-6

[49] Catania A., C. Lonati A. Sordi, andGatti S (2009). Detrimental consequences of brain injury on peripheral cells. Brain Behav. Immun. 23, 877–884 19394418 10.1016/j.bbi.2009.04.006

