# Supplementary material for: Alterations in Peripheral Organs following Combined Hypoxemia and Hemorrhagic Shock in a Rat Model of Penetrating Ballistic-Like Brain Injury
Source: J Neurotrauma. 2020 Feb 4;37(4):656–64. doi: 10.1089/neu.2019.6570 (PMC7045350; doi:10.1089/neu.2019.6570)
Supplement: Supplemental data [file Supp_Fig1.pdf]

Supplementary Data

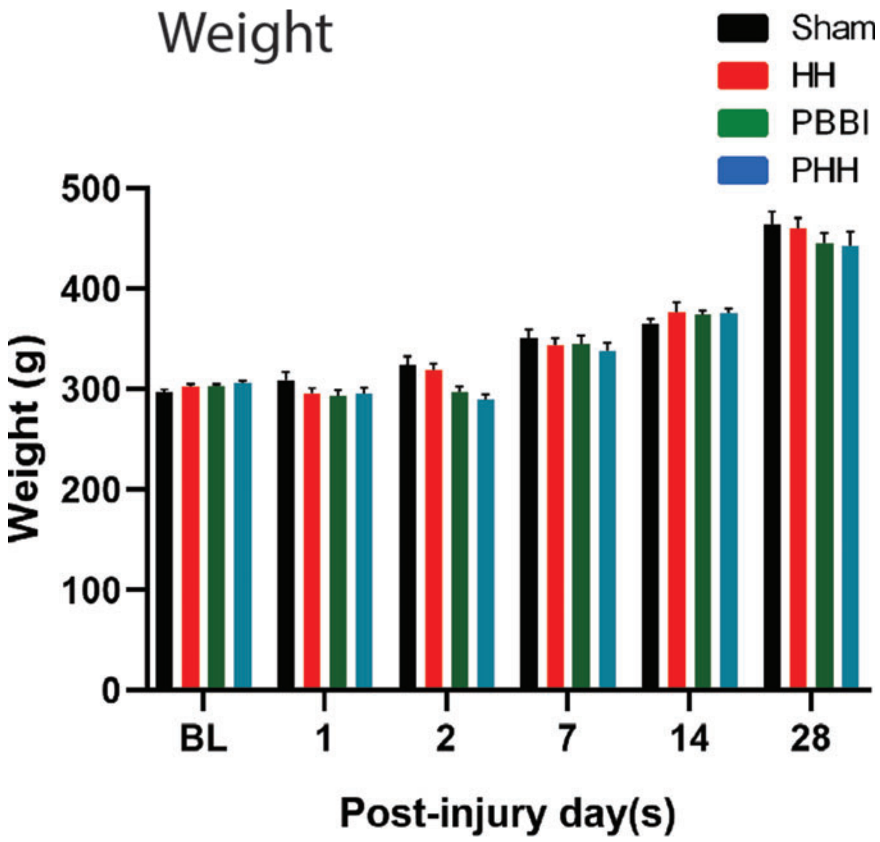

SUPPLEMENTARY FIG. S1. Temporal changes in body weight following injury: Body weight measurements for all the rats in the study showing a non-significant reduction in body weight following penetrating ballistic-like brain injury (PBBI) and polytrauma at 2 days post-injury. Baseline (BL) data is from 50 rats (10 rats from each time-point) per group and for the post-injury set it is $n = 10$ per group per time-point; values are presented as mean ± standard error of the mean. HH, hypoxemia and hemorrhagic shock (HH); PHH, PBBI followed by hypoxemia and hemorrhagic shock.
